# Moyamoya Disease in a 4‐Year‐Old Male Child: A Case Report From Nepal

**DOI:** 10.1002/ccr3.70397

**Published:** 2025-04-15

**Authors:** Pratik Baral, Ishwor Thapaliya, Adesh Kantha, Sandhya Paudel, Ashok Baral

**Affiliations:** ^1^ Institute of Medicine Tribhuvan University Maharajgunj Nepal; ^2^ Gandaki Medical College Pokhara Kaski Nepal; ^3^ Western Regional Hospital Pokhara Kaski Nepal

**Keywords:** antiplatelet therapy, cerebral angiography, internal carotid artery stenosis, Moyamoya disease, recurrent stroke

## Abstract

Moyamoya disease is a rare cerebrovascular disorder characterized by bilateral stenosis or occlusion of internal carotid arteries and abnormal vascular networks. We present a case of a 4‐year‐old child from Nepal with recurrent ischemic strokes, diagnosed with Moyamoya based on the clinical and imaging findings. Management involved conservative therapy with aspirin.

## 1 Introduction

Moyamoya disease (MMD) is a rare, progressive cerebrovascular disorder caused by bilateral stenosis or occlusion of the terminal portion of the internal carotid arteries (ICAs) and/or the proximal portions of the anterior cerebral arteries (ACAs) and middle cerebral arteries (MCAs) [1]. MMD means “puff of smoke” in Japanese and is used to describe the abnormal tangled vascular network compensating for the blockage [2]. It is mostly seen in people living in East Asian countries such as Korea and Japan, compared with those in the Western Hemisphere [3]. It is more common in children and rarely manifests in adults, with a higher incidence in females [4], occurring nearly twice as frequently as in males [5]. MMD demonstrates a bimodal age distribution [5], commonly presenting around 10 years of age and in between 30 and 40 years [6, 7]. It may cause ischemic attacks or cerebral infarction, which occur more frequently in children compared with adults. In adults, cerebral hemorrhage may occur [7].

## 2 Case History/Examination

A 4‐year‐old male child from the Siraha district of Nepal, belonging to a poor socioeconomic family, was brought to the Neurosurgery Out‐Patient Department of TU Teaching Hospital with chief complaints of right‐sided weakness involving upper and lower limbs for around 1 month. The weakness was acute in onset and nonprogressive. The parents reported no history of abnormal body movements, facial deviation, or loss of consciousness. However, they mentioned a similar episode occurring 6 months earlier on the left side, during which the child received conservative treatment elsewhere and was subsequently commenced on aspirin therapy at a dose of 3 mg/kg/day.

On examination, the child's Glasgow Coma Scale (GCS) was 15 (E4V5M6), pupils were bilaterally equal and reactive to light, and there was decreased muscle bulk in both upper and lower limbs (more pronounced on the right side). Power was 3/5 in the right upper limb and 2/5 in the right lower limb, whereas on the left side, it was 4/5 for both upper and lower limbs. Deep tendon reflexes were normal, whereas the plantar reflex was bilaterally upgoing.

## 3 Investigations

Laboratory tests revealed increased prothrombin time (16 s), increased white blood cell count of 15,100/mm^3^, neutropenia (34%, normal range: 45%–75%), lymphocytosis (54%), eosinophilia (10%), low mean cell volume, mean cell hemoglobin, and mean cell hemoglobin concentration, respectively, as shown in Table 1.

**TABLE 1:** Laboratory findings of the patient at the time of presentation.

Laboratory parameters	Results	Units	Reference value
*Complete blood cell count*			
Hemoglobin	12.9	g%	12–16
Packed cell volume (PCV)	40.6	%	36–42
Mean cell volume (MCV)	78	fl	82–92
Mean cell hemoglobin (MCH)	24.7	pg	27–32
Mean cell hemoglobin concentration	31.7	%	32–36
Red blood cells (RBC) count	5.22	millions/cu	
Total leukocyte count (TLC)	15,100	/mm^3^	4000–11,000
Platelet count	371,000	/mm^3^	150,000‐450,000
*Differential count*			
Lymphocytes	54	%	25–45
Neutrophils	34	%	45–75
Monocytes	2	%	2–10
Eosinophils	10	%	1–6
*Coagulation profile*			
Prothrombin time (PT)	16	s	10–13
*Biochemical tests*			
Sodium	139	mEq/L	135–145
Potassium	4.4	mEq/L	3.5–5.5
Random blood sugar	5.7	mmol/L	3.8–7.8
Blood urea	4.6	mmol/L	1.8–6.4
Serum creatinine	30	μmol/L	45–105
*Urine analysis*			
Leucocytes	0–1	/hpf	0–5
Epithelial cells	0–1	/hpf	0–4
*Serological analysis*			
HCV	Negative		
HIV	Non‐reactive		
HBsAg	Negative		

Abbreviation: hpf, high power field.

**FIGURE 1:**
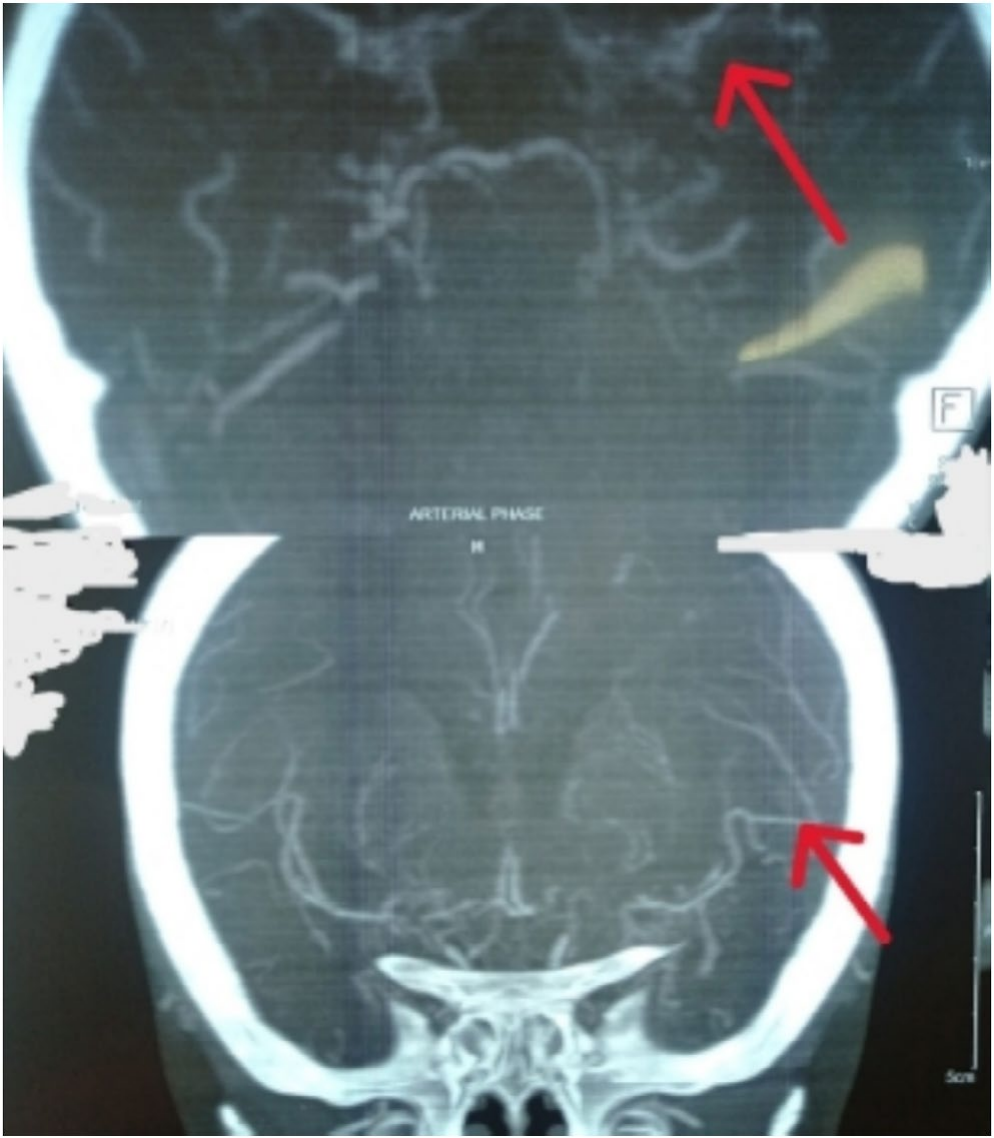
CT scan revealing ill‐defined hypodense area in left fronto‐parietal lobe and right frontal lobe.

Suspecting MMD, a computed tomography (CT) scan of the head with digital subtraction angiography (DSA) of head and neck arteries was performed. The CT scan revealed an ill‐defined hypodense area in the left fronto‐parietal lobe and right frontal lobe, indicative of a subacute infarct in the left MCA territory and chronic infarct in the right MCA territory, respectively, as shown in Figure 1. The scan also showed a reduced caliber of bilateral ICAs from its origin up to the cavernous segment with multiple tortuous collateral vessels supplying the distal anterior circulation from the posterior circulation, in net‐like fashion giving “puff of smoke” appearance around the circle of Willis—features consistent with MMD.

## 4 Treatment and Follow‐Up

On diagnosis, the family received counseling about the potential risks of recurrent strokes and the possibility of poor mental development. Surgical treatment, specifically an extracranial–intracranial bypass procedure, was recommended, but the family declined due to financial constraints. Aspirin was continued, and the patient was on regular follow‐up and physiotherapy training after being discharged. However, the patient showed no signs of improvement during follow‐up visits.

## 5 Discussion

MMD is a progressive vaso‐occlusive disorder in major intracranial arteries resulting in the formation of characteristic collateral vessels [8]. These collaterals are usually tiny, fragile, and highly susceptible to bleeding, aneurysms, and clot formation [9]. The disease mostly affects the anterior circulation and involves bilateral vessels more frequently than unilateral ones [10]. Approximately 40% of cases with initial unilateral presentation subsequently show contralateral involvement [10] as observed in our case. MMD represents around 20%–25% of pediatric strokes attributed to arteriopathy and accounts for 6%–10% of all childhood strokes and transient ischemic attacks (TIAs) [11]. Despite known medical management, it poses a significant risk of recurrence unless treated surgically [12].

The etiology of the disease is still unknown [5]; more than half of children diagnosed with MMD have no identified cause [13]. However, a familial inheritance has been observed in 10%–15% of Japanese cases [14], with transmission occurring in an autosomal recessive fashion [15]. Genome‐wide analysis has identified several gene loci, including 3q24‐p26, 6q25, 8q23, and 17q25, in familial MMD [9, 16]. Moreover, the discovery of p.R4859K as a founder missense mutation in the ring finger protein 213 (RNF213) gene, responsible for RNF213 gene polymorphism, along with its higher carrier frequency in regions such as Japan, may explain the higher prevalence of the disease in East Asia (Japan, Korea, and China) compared with Western countries [17]. It has also been associated with conditions such as Down's syndrome, congenital heart defects, antiphospholipid syndrome, renal artery stenosis, and thyroiditis in the medical literature [8, 13]. In the present case, no identifiable cause was found, and the family history was also inconclusive, suggesting sporadic onset in the patient.

MMD has a low global annual incidence of 0.54 per 100,000 population [18]. Despite being an Asian country, Nepal has reported only four cases of MMD, comprising three adult cases and one pediatric female case [15]. This marks the first reported case of pediatric‐onset MMD in a male child from Nepal. The exact cause for its rare occurrence in the country still remains unknown [15].

The most prevalent manifestation of MMD in children is ischemic stroke (80%) rather than hemorrhage (20%) [18], secondary to cerebral hypoperfusion due to progressive occlusion of major vessels [3]. Children with MMD commonly experience repeated TIAs or infarctions in the territory of the ICA, particularly in the frontal lobe, often presenting with unilateral muscular weakness, paralysis, and seizures [19]. Our case now presents with right‐sided weakness and has a preceding history of right‐sided ischemic stroke 6 months earlier. Other symptoms may include headache, fever, blindness, aphasia, or focal neurological deficits [10, 13]; however, these are absent in our case. In contrast, adults typically suffer from subarachnoid or intraparenchymal hemorrhage [5].

DSA of cerebral arteries is considered as the gold standard for diagnosis of MMD [20]. DSA of cerebral arteries in our case revealed bilateral ICA narrowing with tortuous collateral vessels, resulting in a “puff of smoke” appearance around the circle of Willis as described in the existing literature on MMD [7, 17, 20]. Head CT scans commonly reveal hypodense areas indicative of infarction in cortical watershed zones, basal ganglia, deep white matter, or periventricular regions as observed in our case [21]. However, due to its unknown etiology, other conditions with similar angiographic findings, such as atherosclerosis, autoimmune disorders, meningitis, brain tumors, Down syndrome, von Recklinghausen's disease, head trauma, and post‐radiation state, must be ruled out before confirming the diagnosis [7]. Thus, diagnosing MMD can be challenging but should be guided by its characteristic presentation, underlying pathology, and appropriate angiography studies [8].

Acute management of MMD primarily focuses on symptomatic relief, aiming to reduce elevated intracranial pressure, enhance cerebral blood flow, and manage seizures [13, 22]. Medical management mainly involved antiplatelet therapy given to prevent recurrent thrombosis [19]. Indirect bypass surgery, such as encephaloduroarteriosynangiosis, is often preferred in children due to their vessels being too small for direct anastomosis [20]. These procedures aim to restore blood flow to hypoxic brain tissue by reopening occluded blood vessels [19]. However, indirect bypass surgery may lead to complications such as postoperative headache due to poorer collateral circulation compared to direct procedures [23].

Affording surgical treatment options is often challenging in low‐ and middle‐income countries such as Nepal due to their high costs. Although surgical modalities are preferred over conservative approaches, a nationwide survey by Gupta and colleagues found an unmet surgical need of 10%, with head, neck, and face surgeries constituting the largest portion at 31.8% [24]. Another survey on barriers to surgical care in Nepal found that about 20% of individuals needing surgery did not receive it due to lack of affordability [25]. The issue of affordability becomes more prevalent in populations with low socioeconomic status and living in rural areas. With an estimated 23% of deaths being preventable with adequate surgical care, there is a great need for support for patients to overcome these financial barriers [24].

Thus, aspirin is recommended as initial therapy for pediatric acute anterior ischemic stroke secondary to MMD over no treatment in resource‐limited settings like ours [11, 17]. Patients with MMD should take aspirin daily to prevent ischemic symptoms resulting from microthrombus formation, with dosages ranging from 80 mg/day in children under 6 years to up to 300 mg/day in adolescents, adjusted based on side effects such as easy bruising or gastrointestinal bleeding [21]. However, it is crucial to note that aspirin on long‐term use may increase the risk of intracranial hemorrhage, especially in patients with choroidal collaterals [17, 26].

## 6 Conclusion

MMD should be considered in the differential diagnosis when children present with signs and symptoms of acute ischemic stroke even in regions with rare occurrence. This case highlights the challenges of managing MMD in resource‐limited settings like Nepal, where financial constraints often dictate treatment decisions. A nonsurgical approach, such as aspirin and physiotherapy, may offer an effective alternative to no treatment in such settings.

## Author Contributions

**Pratik Baral:** writing – original draft, writing – review and editing. **Ishwor Thapaliya:** writing – original draft, writing – review and editing. **Adesh Kantha:** writing – review and editing. **Sandhya Paudel:** writing – review and editing. **Ashok Baral:** writing – review and editing.

## Ethics Statement

Our institution does not require ethical approval for reporting individual cases or case series.

## Consent

As the patient is a minor subject, written informed consent was obtained from the patient's parent to publish this report as per the journal's patient consent policy.

## Conflicts of Interest

The authors declare no conflicts of interest.

## Data Availability

All the data generated during this study can be accessed through direct communication with the corresponding author and the agreement of all research team members.
